# Improved control of SARS-CoV-2 by treatment with a nucleocapsid-specific monoclonal antibody

**DOI:** 10.1172/JCI162282

**Published:** 2022-12-01

**Authors:** Tanushree Dangi, Sarah Sanchez, Jacob Class, Michelle Richner, Lavanya Visvabharathy, Young Rock Chung, Kirsten Bentley, Richard J. Stanton, Igor J. Koralnik, Justin M. Richner, Pablo Penaloza-MacMaster

**Affiliations:** 1 Department of Microbiology and Immunology, Northwestern University Feinberg School of Medicine, Chicago, Illinois, USA.; 2 Department of Microbiology and Immunology, University of Illinois College of Medicine at Chicago, Chicago, Illinois, USA.; 3 Ken and Ruth Davee Department of Neurology, Feinberg School of Medicine, Northwestern University, Chicago, Illinois, USA.; 4 Division of Infection and Immunity, School of Medicine, Cardiff University, Cardiff, United Kingdom.

**Keywords:** COVID-19, Immunology, Adaptive immunity

## Abstract

The severe acute respiratory syndrome coronavirus 2 (SARS-CoV-2) spike protein is the main antigen in all approved COVID-19 vaccines and is also the only target for monoclonal antibody (mAb) therapies. Immune responses to other viral antigens are generated after SARS-CoV-2 infection, but their contribution to the antiviral response remains unclear. Here, we interrogated whether nucleocapsid-specific antibodies can improve protection against SARS-CoV-2. We first immunized mice with a nucleocapsid-based vaccine and then transferred sera from these mice into naive mice, followed by challenge with SARS-CoV-2. We show that mice that received nucleocapsid-specific sera or a nucleocapsid-specific mAb exhibited enhanced control of SARS-CoV-2. Nucleocapsid-specific antibodies elicited NK-mediated, antibody-dependent cellular cytotoxicity (ADCC) against infected cells. To our knowledge, these findings provide the first demonstration in the coronavirus literature that antibody responses specific to the nucleocapsid protein can improve viral clearance, providing a rationale for the clinical evaluation of nucleocapsid-based mAb therapies to treat COVID-19.

## Graphical abstract

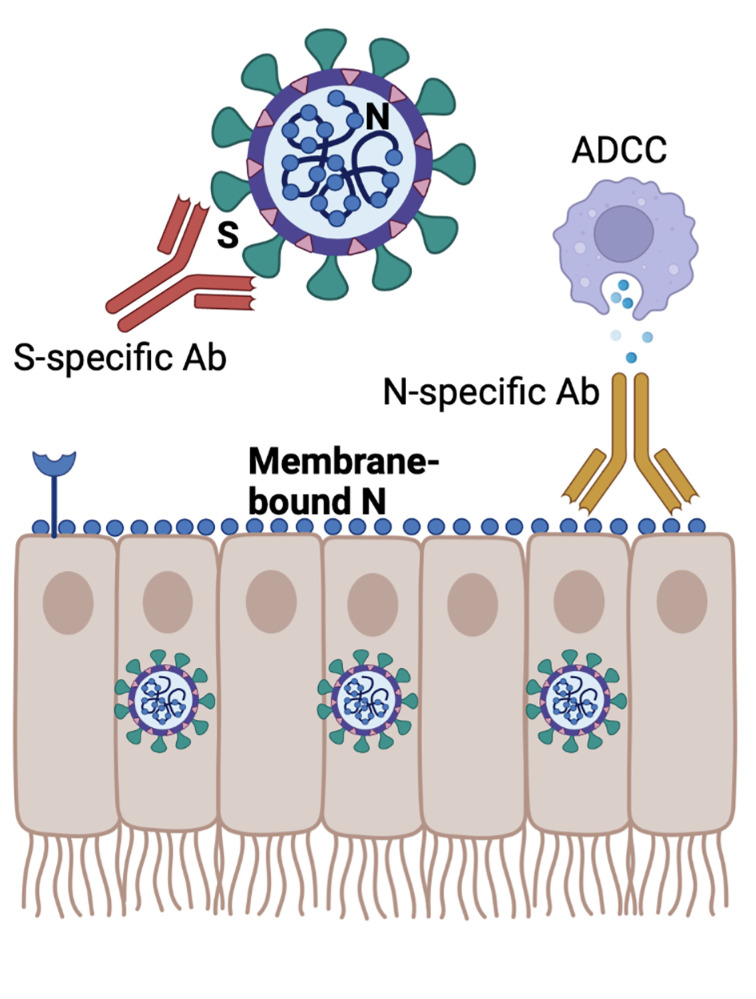

## Introduction

Severe acute respiratory syndrome coronavirus 2 (SARS-CoV-2) has infected more than 600 million people and continues to spread around the globe. Although vaccines and monoclonal antibody (mAb) therapies can prevent severe disease and death, breakthrough infections can occur, highlighting the need to improve current vaccines and available treatments (1–9). The SARS-CoV-2 spike protein is critical for virus entry, making this protein an important antigen present in SARS-CoV-2 vaccines and the only target for all mAb therapies. Besides spike-specific immune responses, other antigen-specific immune responses are elicited during SARS-CoV-2 infection (10–13), but their role in protecting against infection remains unclear.

In particular, it is unknown whether antibodies specific to internal viral proteins such as the nucleocapsid protein, which does not play a role in virus entry, can confer protection against SARS-CoV-2. Knowing whether other antigen-specific antibodies are protective could facilitate the development of more potent vaccines and mAb therapies for coronavirus infections. In this study, we studied nucleocapsid-specific immune responses in a cohort of patients with COVID-19 and interrogated whether nucleocapsid-specific antibody responses elicited by a novel nucleocapsid-based vaccine could confer protection against a SARS-CoV-2 challenge in K18-hACE2 mice. Interestingly, we found that nucleocapsid-specific humoral responses and a nucleocapsid-specific mAb could mediate antibody-dependent cellular cytotoxicity (ADCC) and help control SARS-CoV-2 infection when given as pre-exposure prophylaxis. Together, these data warrant the clinical evaluation of nucleocapsid-specific mAb therapies for the treatment of SARS-CoV-2 and suggest that the inclusion of the nucleocapsid protein in next-generation vaccines could confer an additional immunological benefit.

## Results

### Adaptive immune responses elicited by a nucleocapsid vaccine help control a SARS-CoV-2 infection.

**Figure 1:**
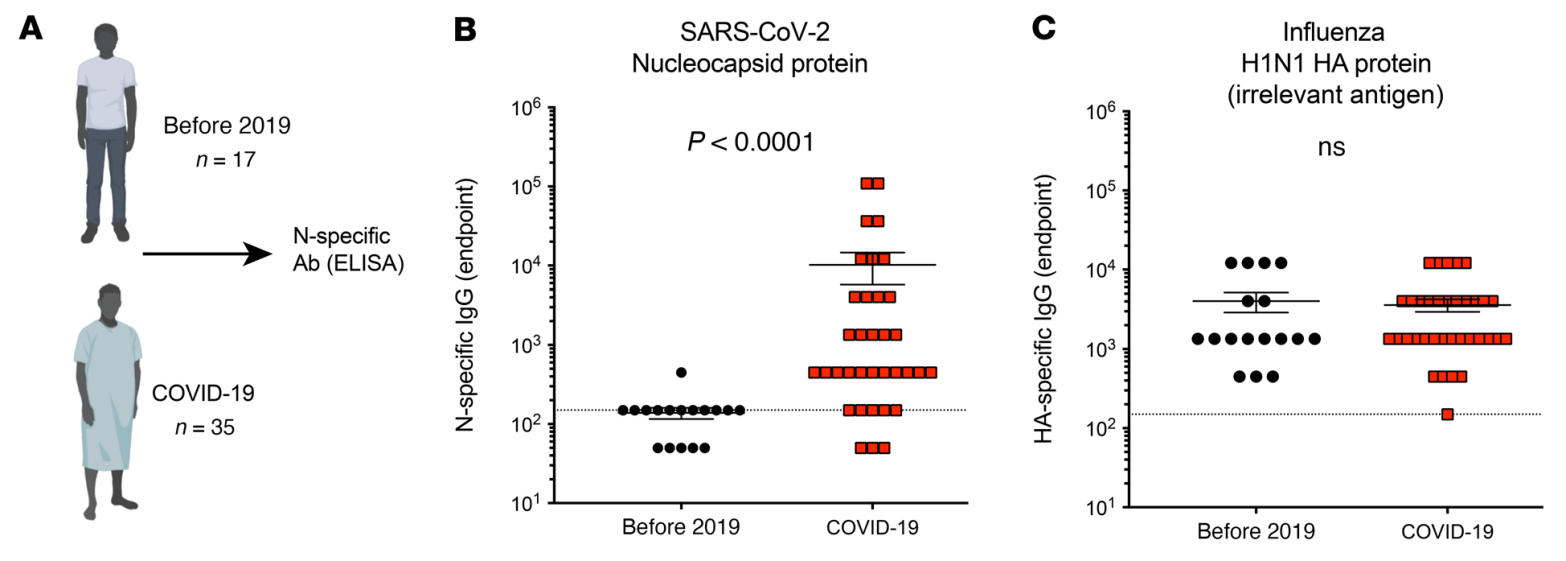
SARS-CoV-2 nucleocapsid–specific antibody after SARS-CoV-2 infection in a cohort of patients admitted to Northwestern University Hospital. (**A**) Human pre-2019 plasma samples from healthy individuals were used as a control. Data shown are from an ongoing study, in which participants were infected on different dates, hence the heterogeneity in the nucleocapsid-specific antibody responses. SARS-CoV-2 infection was confirmed by RT-PCR. Antibody responses were evaluated by ELISA. (**B**) Summary of SARS-CoV-2 nucleocapsid–specific antibodies in sera. (**C**) Summary of influenza HA–specific antibodies in sera (used as an irrelevant antigen control). Dashed lines represent the LOD. Significance in **B** and **C** was determine by Mann-Whitney *U* test. Error bars represent the SEM.

All approved COVID-19 vaccines express the spike protein of SARS-CoV-2. Immune responses against other antigens, for example against the nucleocapsid antigen, are not elicited after SARS-CoV-2 vaccination but can be induced after natural SARS-CoV-2 infection. As shown in Figure 1, A and B, we detected nucleocapsid-specific antibody responses in the plasma of patients with COVID-19, but not in the plasma of individuals before the 2019 pandemic. We detected similar antibody responses against an irrelevant viral antigen (influenza) in SARS-CoV-2–exposed and –unexposed individuals (Figure 1C). Although patients with COVID-19 show nucleocapsid-specific immune responses, it is still unclear whether nucleocapsid-specific immune responses can play an antiviral role in vivo. In particular, it is unknown whether antibodies against nucleocapsid (an internal viral protein that is not a target of neutralization) could have an effect during a SARS-CoV-2 infection.

**Figure 2:**
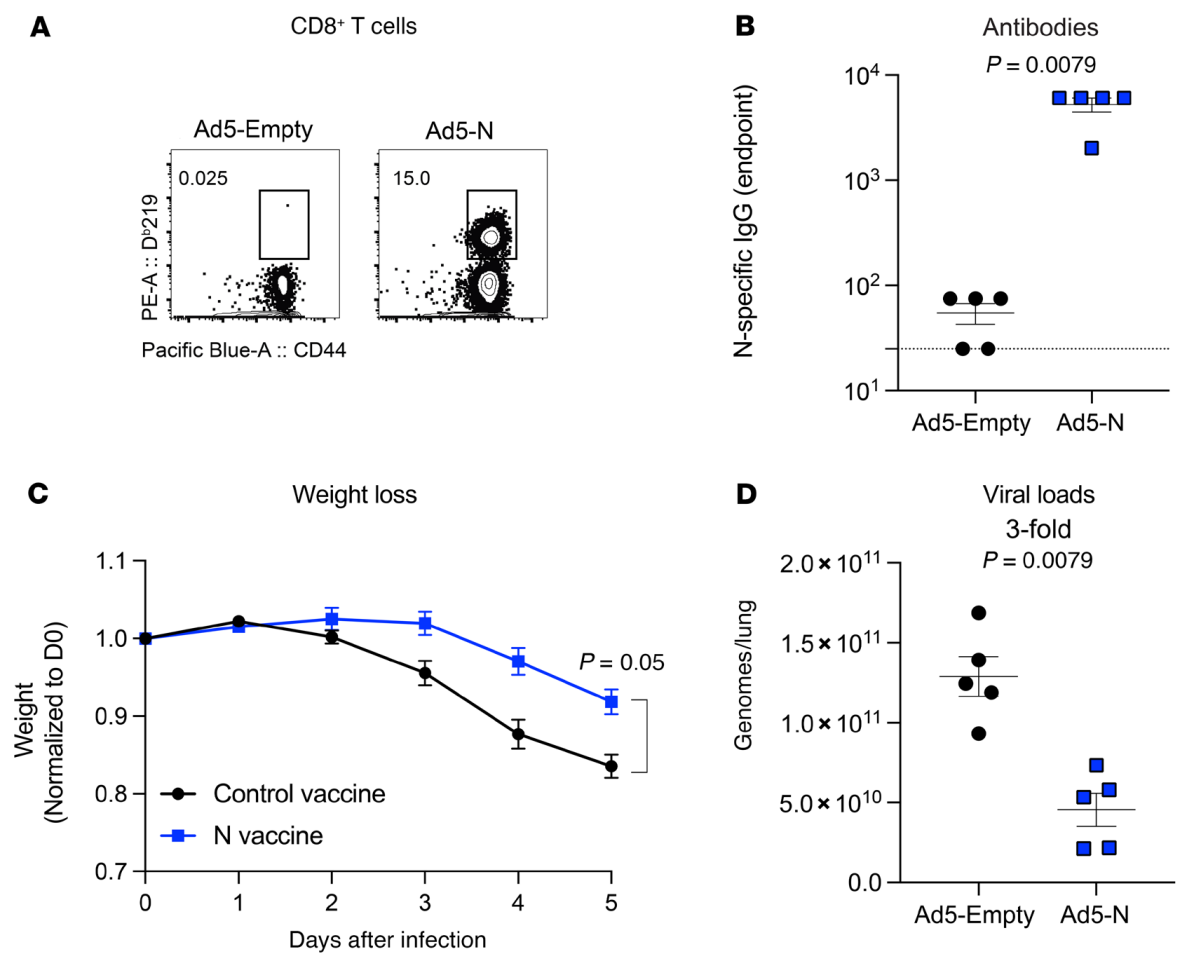
A nucleocapsid vaccine improves the control of SARS-CoV-2 infection. (**A**) Representative FACS plots showing the frequencies of SARS-CoV-2 nucleocapsid–specific CD8^+^ T cells (D^b^N219^+^) in PBMCs. (**B**) Summary of SARS-CoV-2 nucleocapsid–specific antibodies in sera. (**C**) Weight loss following infection. (**D**) Viral loads in lungs on day 5 after infection. K18-hACE2 mice were primed intramuscularly with Ad5-N, and after 2 weeks adaptive immune responses were evaluated, and mice were challenged intranasally with 5 × 10^4^ PFU of SARS-CoV-2. Viral RNA was quantified by RT-qPCR. Challenges were performed with a total of 5 mice per group in BSL-3 facilities. *P* values were calculated using a Mann-Whitney *U* test. Error bars represent the SEM.

We previously showed that a nucleocapsid-based vaccine does not confer significant protection against an intranasal SARS-CoV-2 challenge when given as a “single vaccine,” without a spike-based vaccine (14). In that prior report, we evaluated viral loads at a very early point after infection (day 3 after infection) to measure breakthrough infection. In our follow-up studies, we evaluated viral control at later points after infection. We vaccinated K18-hACE2 mice intramuscularly with an adenovirus serotype 5 vector expressing SARS-CoV-2 nucleocapsid (Ad5-N) at a dose of 10^11^ PFU per mouse. We used K18-hACE2 mice because they are susceptible to SARS-CoV-2 and are widely used to evaluate vaccines (14–19). Two weeks after vaccination, we detected nucleocapsid-specific CD8^+^ T cell responses (Figure 2A) and antibody responses (Figure 2B) in mice vaccinated with the nucleocapsid vaccine. Then we challenged these K18-hACE2 mice intranasally with a high dose (4 × 10^5^ PFU) of SARS-CoV-2 (isolate USA-WA1/2020), and on post-challenge day 5, we quantified the viral loads in lungs by reverse transcription quantitative PCR (RT-qPCR). Interestingly, the mice that received the nucleocapsid vaccine showed significantly lower weight loss (Figure 2C) and lower viral loads (Figure 2D) compared with the control vaccinated mice. Altogether, our prior study showed that a nucleocapsid vaccine does not prevent breakthrough infection and does not significantly reduce viral titers during the hyperacute phase (day 3 after infection) (14), but our new data in Figure 2D show that a nucleocapsid vaccine may facilitate viral control at later time points (day 5 after infection).

Nucleocapsid-specific T cell responses have been suggested to contribute to viral control following SARS-CoV-2 infection (20, 21), but the role of nucleocapsid-specific antibody responses remains elusive (since nucleocapsid-specific antibodies do not target a neutralizing epitope on SARS-CoV-2).

### Immunogenicity of a nucleocapsid prime-boost vaccine regimen.

**Figure 3:**
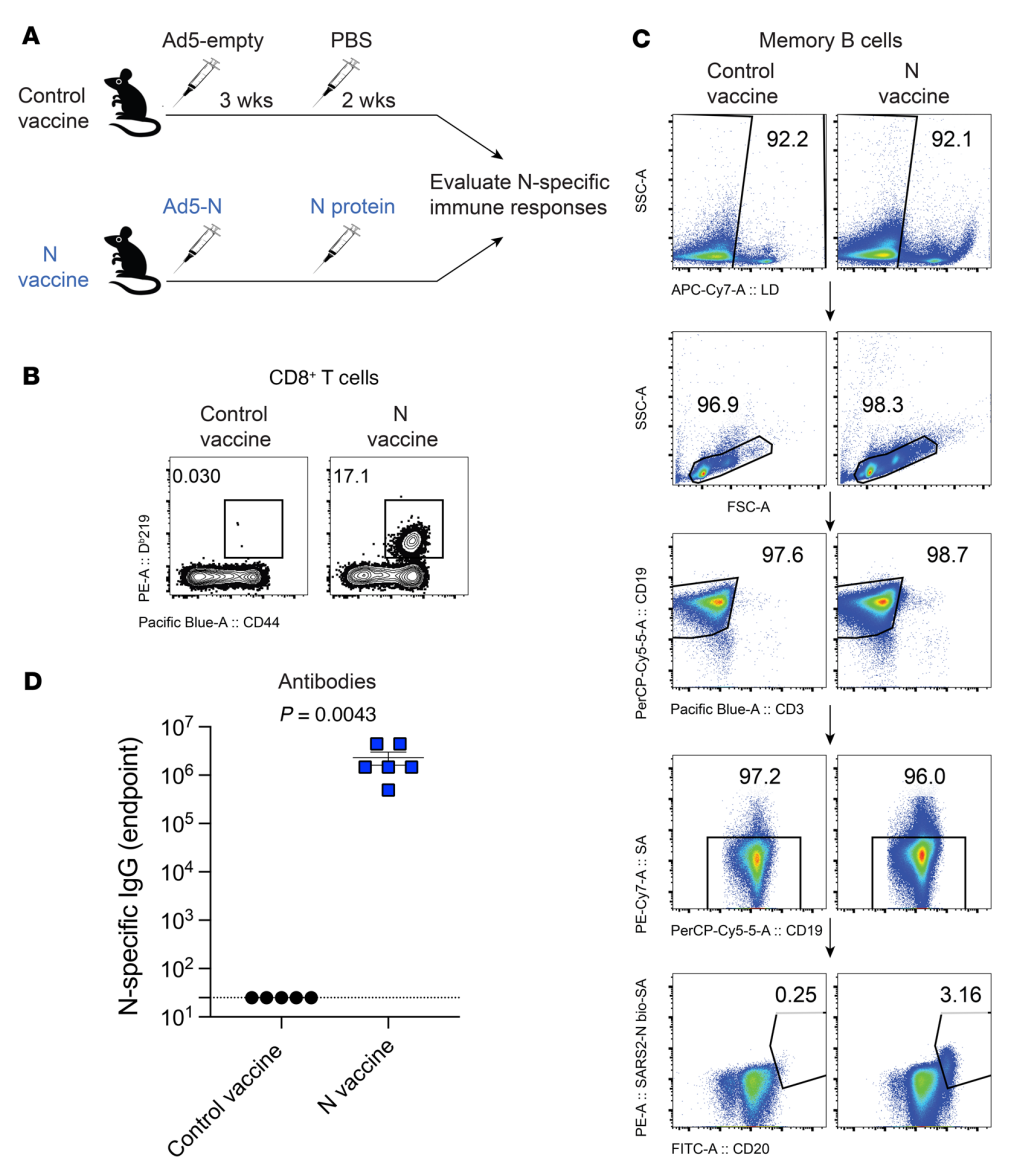
Immunogenicity of a SARS-CoV-2 nucleocapsid vaccine regimen. (**A**) Experimental approach for evaluating immune responses after nucleocapsid vaccination. (**B**) Representative FACS plots showing the frequencies of SARS-CoV-2 nucleocapsid–specific CD8^+^ T cells (D^b^N219^+^) in PBMCs. (**C**) Representative FACS plots showing the frequencies of SARS-CoV-2 nucleocapsid–specific memory B cells in spleen. Splenocytes were MACS purified by negative selection to enrich for B cells, facilitating the visualization of nucleocapsid-specific B cells. SSC-A, side scatter area. (**D**) Summary of SARS-CoV-2 nucleocapsid–specific antibodies in sera. Data are from week 2 after the boost and are from 1 experiment. *n* = 5–6 per group. The experiment was performed twice with similar results. The *P* value was calculated using a Mann-Whitney *U* test. Error bar represents the SEM.

To understand the role of nucleocapsid-specific humoral responses during SARS-CoV-2 infection, we developed a prime-boost vaccine regimen that elicited high levels of nucleocapsid-specific antibodies, which were later used in passive immunization experiments. We primed C57BL/6 mice intramuscularly with Ad5-N (14, 21, 22) at a dose of 10^11^ PFU per mouse, followed by a booster with 100 μg nucleocapsid protein 3 weeks later to generate high titers of nucleocapsid-specific antibody responses. As controls, we immunized mice with an “empty” Ad5 vector (Ad5-empty) followed by PBS boost. Two weeks after the boost, we measured nucleocapsid-specific immune responses (Figure 3A). Mice immunized with the nucleocapsid vaccine regimen exhibited robust nucleocapsid-specific CD8^+^ T cell responses (Figure 3B), memory B cell responses (Figure 3C), and antibody responses (Figure 3D).

**Figure 4:**
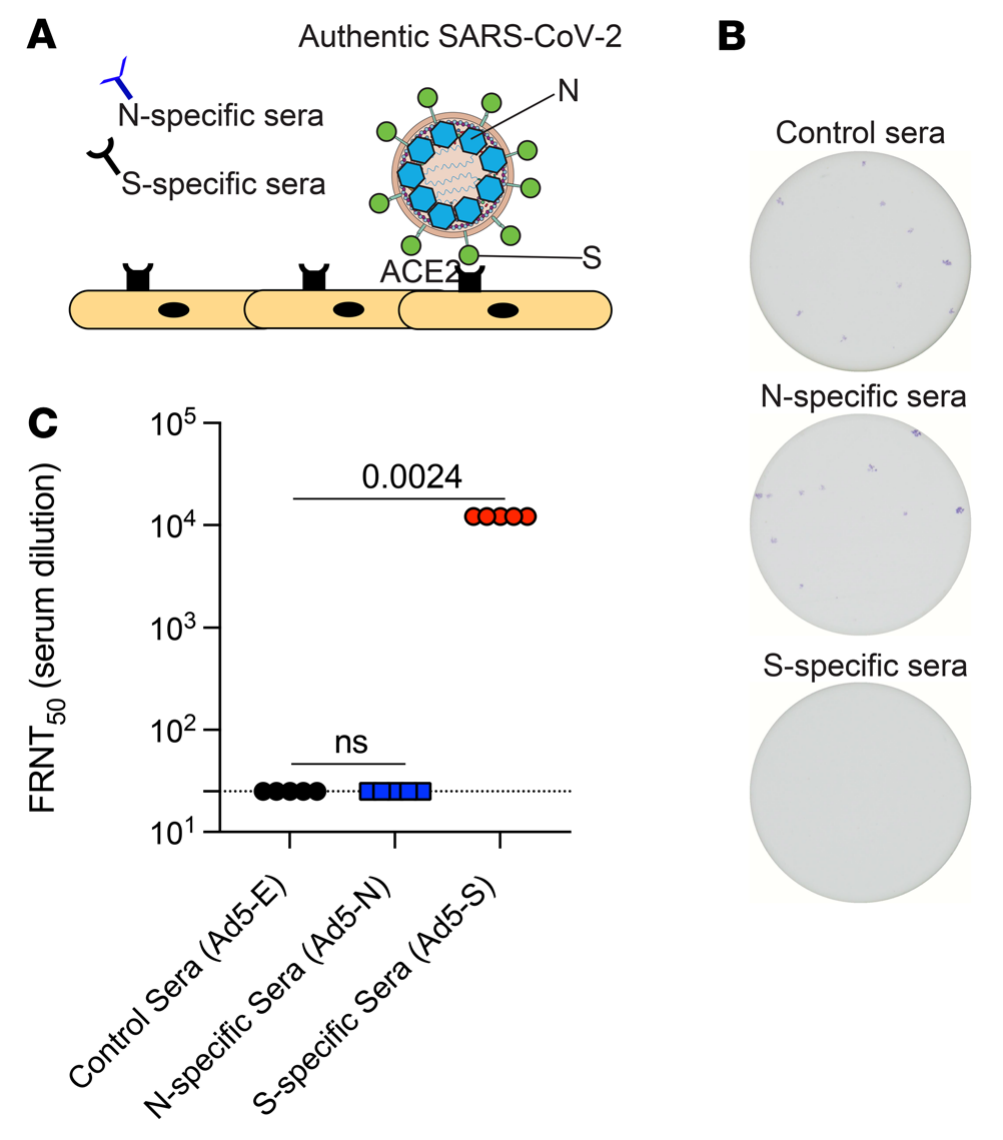
Nucleocapsid-specific humoral responses do not prevent SARS-CoV-2 infection. (**A**) Experimental approach for performing FRNT assays on Vero cells using live SARS-CoV-2 (USA-WA1/2020). See Methods for technical information. (**B**) Representative wells showing the frequencies of SARS-CoV-2^+^ cells (1:450 sera dilution). (**C**) Summary of FRNT_50_ titers in sera. Ad5-E, Ad5-empty (control). Data are from week 3 after vaccination and are from 1 experiment. *n* = 5 per group. The experiment was performed twice with similar results. Significance was determined using the Kruskal-Wallis test (for multiple comparisons).

Prior studies have suggested that nucleocapsid-specific T cells can help clear SARS-CoV-2 infection via the killing of infected cells (14, 20–22). However, it is still unclear whether nucleocapsid-specific antibodies play any role in antiviral control, since the nucleocapsid is an internal viral protein that is not involved in virus entry. We performed focus reduction neutralization titer (FRNT) assays using live SARS-CoV-2 to determine whether nucleocapsid-specific antibodies prevent SARS-CoV-2 infection (Figure 4A). We used live SARS-CoV-2 instead of a pseudovirus, because live virus contains all viral proteins, including nucleocapsid proteins. As a positive control, we used sera from mice that received a spike-based adenovirus vaccine (Ad5-S, similar to the CanSino vaccine and the Sputnik vaccine). As expected, based on prior studies (22, 23), sera from mice that received the spike-based vaccine completely prevented SARS-CoV-2 infection, even when the sera were diluted 450-fold (Figure 4B). However, sera from mice that received the nucleocapsid-based vaccine did not exert any antiviral effect in this in vitro infection assay (Figure 4, B and C). Taken together, we found that only spike-specific (and not nucleocapsid-specific) antibodies could block SARS-CoV-2 infection, consistent with the widely established notion that spike-specific antibodies are protective, as they can block the first step in the SARS-CoV-2 life cycle: entry into host cells.

### Nucleocapsid-specific antibodies help clear a SARS-CoV-2 infection in vivo.

**Figure 5:**
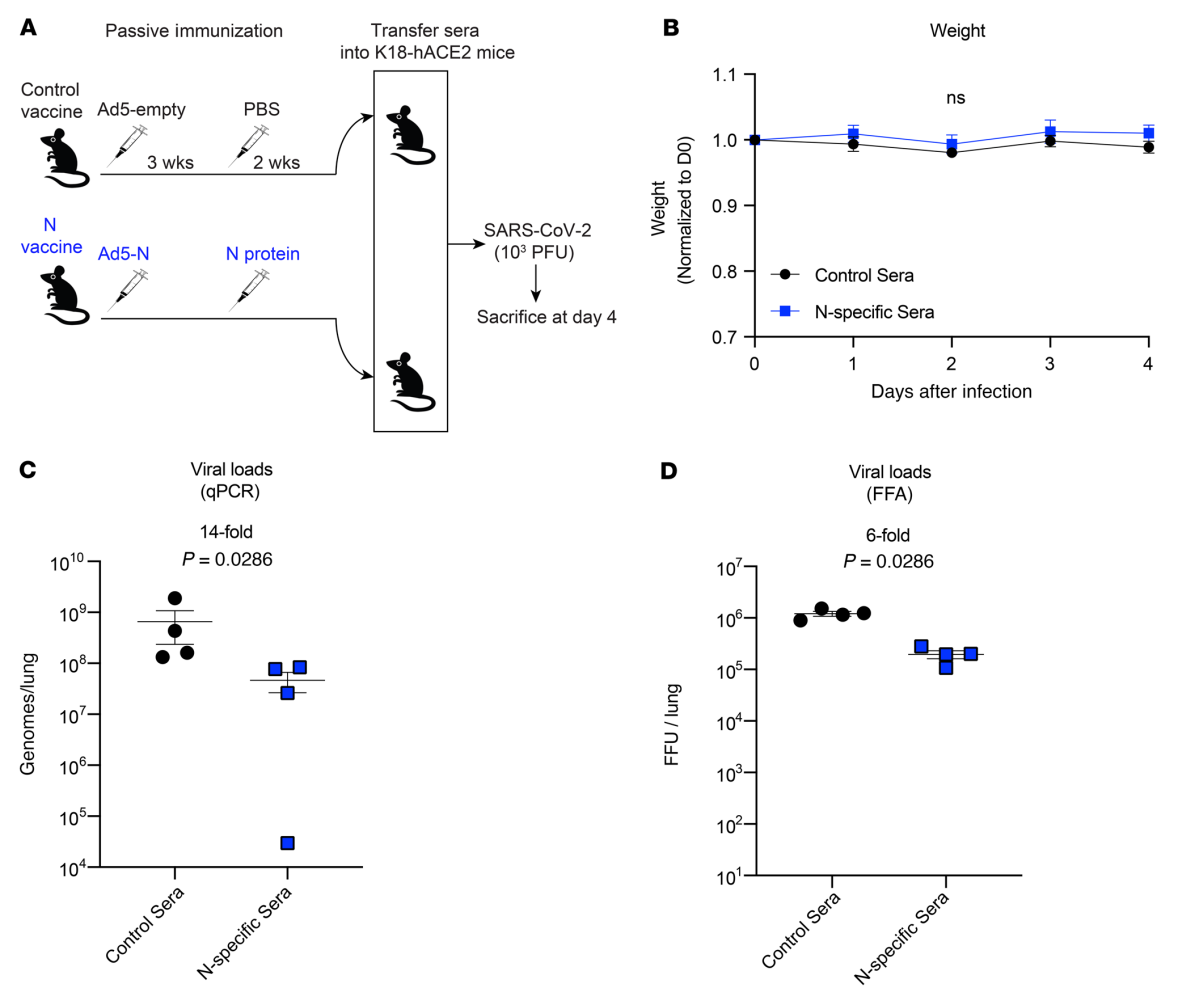
Nucleocapsid-specific humoral responses improve the control of SARS-CoV-2 infection. (**A**) Experimental approach for evaluating viral control after passive immunization with nucleocapsid-specific sera. (**B**) Weight loss following infection. (**C**) Viral loads in lungs as determined by RT-pPCR. (**D**) Viral loads in lungs as assessed by FFAs. C57BL/6 mice were primed intramuscularly with Ad5-N, and after 3 weeks they were boosted with soluble nucleocapsid (N) protein. Two weeks after the boost, sera from these mice were pooled, and 500 μL of these sera were adoptively transferred into naive K18-hACE2 mice. On the following day, the K18-hACE2 mice were challenged intranasally with 10^3^ PFU SARS-CoV-2. RNA was harvested from the lungs on post-infection day 4, and viral RNA was quantified. Challenges were performed with a total of 4 mice per group in BSL-3 facilities. *P* values were calculated using the Mann-Whitney *U* test. Error bars represent the SEM.

Antibody responses exert antiviral functions by various mechanisms, including virus neutralization and Fc-dependent effector mechanisms. Although nucleocapsid-specific sera did not prevent SARS-CoV-2 infection in vitro, we reasoned that it could confer protection in vivo via effector mechanisms. To test this hypothesis, we performed a passive immunization study to evaluate whether the transfer of nucleocapsid-specific antibodies could confer a clinical benefit (Figure 5A). We adoptively transferred 500 μL nucleocapsid-specific sera (week 2 after the boost; same mice from Figure 3A) into naive K18-hACE2 mice. One day after sera transfer, we challenged these K18-hACE2 mice intranasally with a low dose (10^3^ PFU) of SARS-CoV-2, followed by evaluation of weight loss and viral loads on post-challenge day 4. Following this low-dose viral challenge, we did not observe weight loss in the mice (Figure 5B). However, the mice that received nucleocapsid-immune sera showed a significant reduction in viral titers relative to the control mice, as determined by RT-qPCR and focus-forming assays (FFAs) (Figure 5, C and D). These findings demonstrate that even though nucleocapsid-specific humoral responses did not neutralize SARS-CoV-2 in vitro, they could exert antiviral effects in vivo.

**Figure 6:**
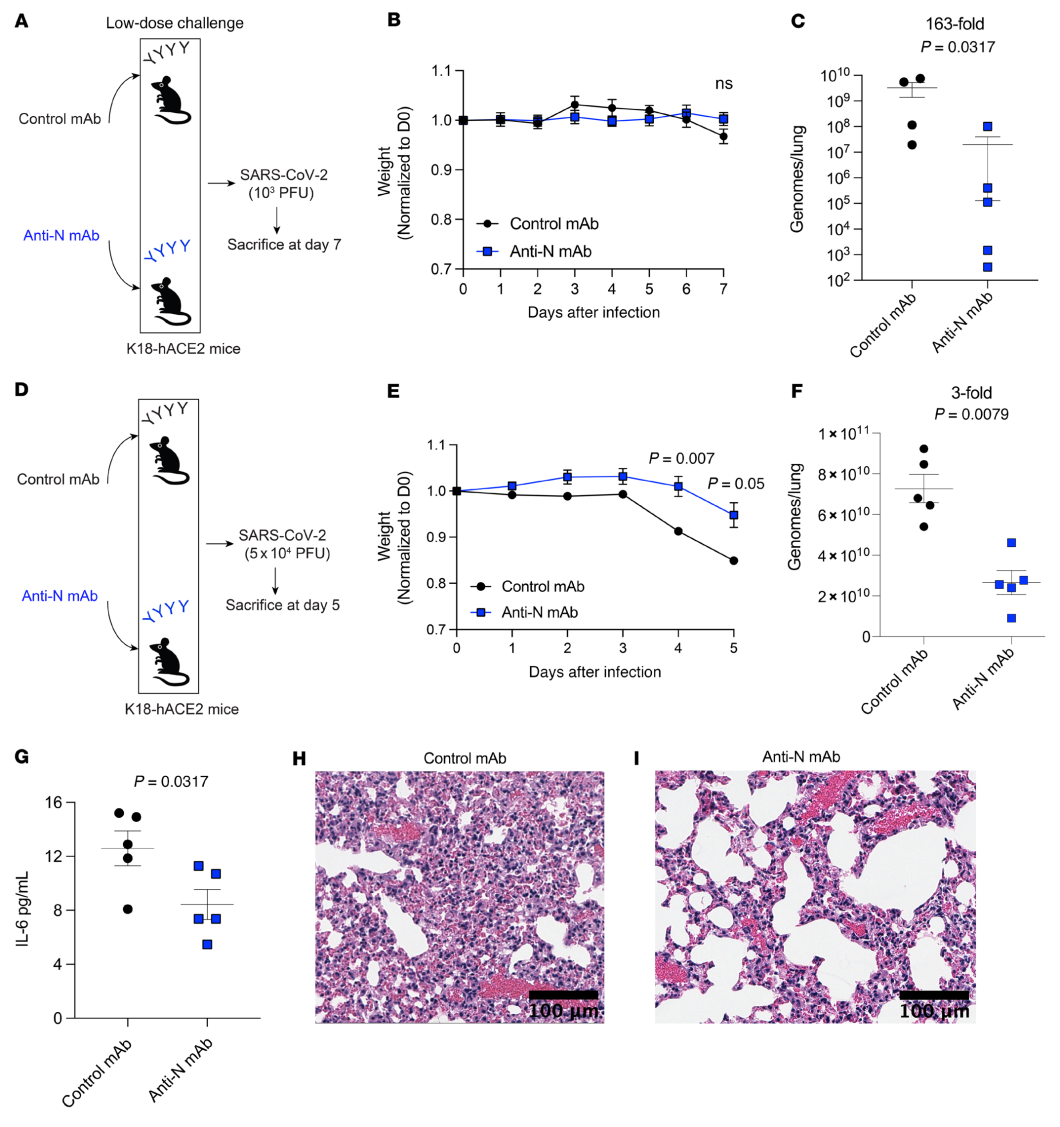
Nucleocapsid-specific mAb improves the control of SARS-CoV-2 infection. (**A**) Experimental approach for evaluating viral control after treatment with a nucleocapsid-specific mAb during a low-dose viral challenge. mAbs (800 μg, IgG control or anti-N) were injected intraperitoneally into naive K18-hACE2 mice. On the following day, the K18-hACE2 mice were challenged intranasally with 10^3^ PFU SARS-CoV-2. (**B**) Weight loss following infection. (**C**) Viral loads in lungs as determined by RT-qPCR. RNA was harvested from the lungs on post-infection day 7, and viral RNA was quantified. (**D**) Experimental approach for evaluating viral control after treatment with a nucleocapsid-specific mAb during a high-dose viral challenge. mAbs (800 μg, IgG control or anti-N) were injected intraperitoneally into naive K18-hACE2 mice. On the following day, the K18-hACE2 mice were challenged intranasally with 5 × 10^4^ PFU SARS-CoV-2. (**E**) Weight loss following infection. (**F**) Viral loads in lungs as determined by RT-qPCR. RNA was harvested from the lungs on post-infection day 5, and viral RNA was quantified. (**G**) IL-6 levels in sera. (**H** and **I**) H&E-stained images of lung. Scale bars: 100 μm. Data in **A**–**C** are from low-dose viral challenges, and data in **D**–**I** are from high-dose viral challenges. Challenges were performed with a total of 4–5 mice per group in BSL-3 facilities. *P* values were calculated using a Mann-Whitney *U* test. Error bars represent the SEM.

The studies above suggest that nucleocapsid-specific antibodies can help clear a SARS-CoV-2 infection. However, sera contain other molecules, such as complement and cytokines, that could mediate antiviral control besides antibodies. To ascertain the specific contribution of nucleocapsid-specific antibody, we treated K18-hACE2 mice with a nucleocapsid-specific mAb or an isotype control antibody, and on the next day, mice received a low-dose intranasal SARS-CoV-2 challenge (10^3^ PFU) followed by evaluation of viral loads in lungs on day 7 after infection (Figure 6A). With this low-dose viral challenge, there was no weight loss by day 7 (Figure 6B). Consistent with our prior results, treatment with a nucleocapsid-specific mAb resulted in a significant improvement in viral control by RT-qPCR (163-fold lower viral titers, relative to control) (Figure 6C). We also observed improved antiviral control in the mice treated with nucleocapsid-specific mAbs by another virological quantification method, FFA (Supplemental Figure 1; supplemental material available online with this article; https://doi.org/10.1172/JCI162282DS1). In a separate experiment, we quantified lung viral loads at a more acute time point (day 4) and observed a similar improvement in viral control in mice that received the nucleocapsid-specific mAb (Supplemental Figure 2). We did not observe statistically significant differences in CD8^+^ T cell responses (Supplemental Figure 3).

We also performed viral challenges using a high dose of SARS-CoV-2 (5 × 10^4^ PFU) that normally results in severe weight loss and immunopathology (Figure 6D). We sacrificed mice on day 5 after infection, because this high viral challenge dose causes severe pneumonia and lethality within approximately 1 week of challenge. Interestingly, treatment with a nucleocapsid-specific mAb mitigated weight loss (Figure 6E) and resulted in a significant improvement in viral control (Figure 6F). In addition, mice treated with a nucleocapsid-specific mAb had lower levels of inflammatory IL-6 cytokine in circulation (Figure 6G) and showed reduced lung immunopathology (Figure 6, H and I) relative to control mice. Altogether, these data demonstrate that nucleocapsid-specific antibodies contribute to the control of a SARS-CoV-2 infection and can mitigate COVID-19 disease progression.

**Figure 7:**
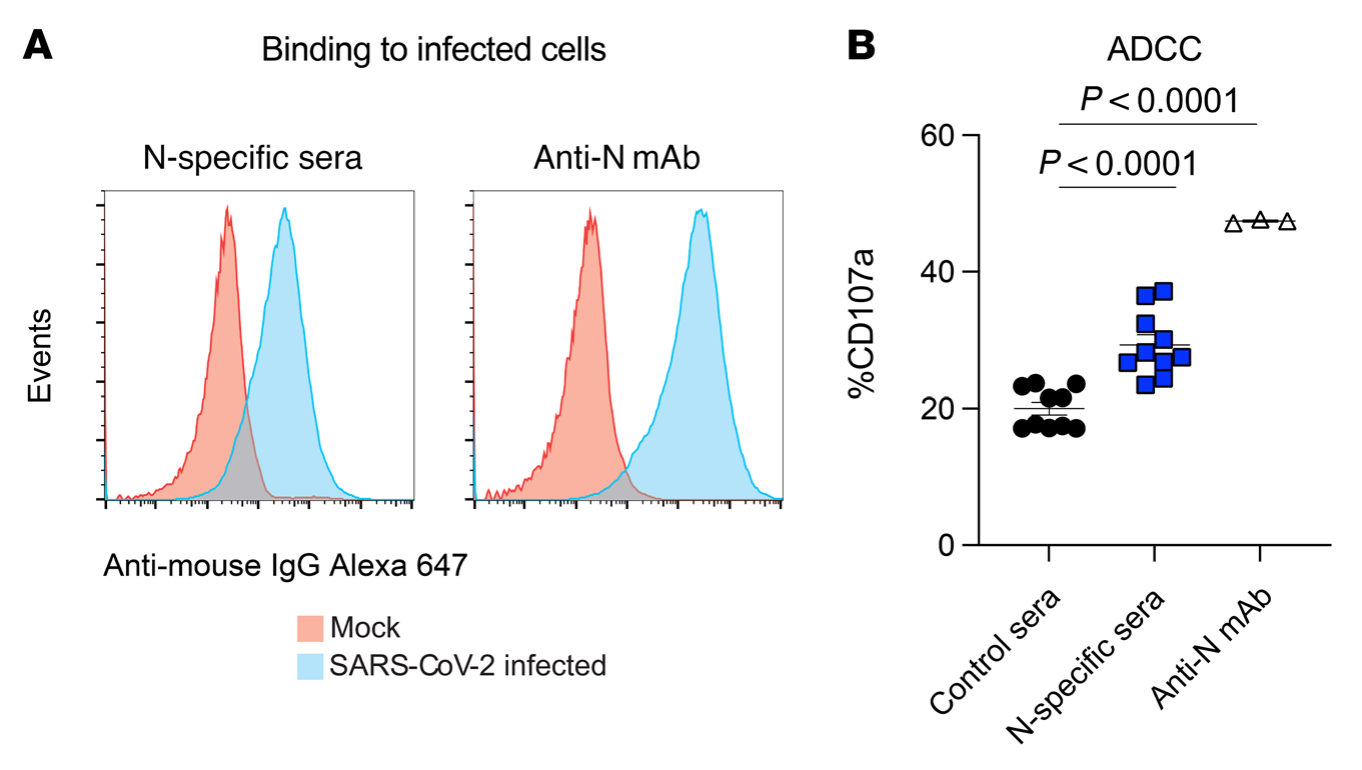
N-specific antibodies bind to infected cells and trigger ADCC. (**A**) Representative FACS plots showing that N-specific sera and the N-specific mAb bound to SARS-CoV-2 infected cells. 293-ACE2 cells were infected with SARS-CoV-2, and binding to N-specific antibody was assessed by flow cytometry using a secondary antibody bound to Alexa Fluor 647. Control cells were mock infected. (**B**) N-specific antibodies triggered ADCC. 293-ACE2 cells were infected with SARS-CoV-2 and cocultured with effector cells (NKL-mCD16 cells), together with N-specific immune sera or anti-N mAb (clone 1C7). CD107a expression on NKL-mCD16 cells was quantified by flow cytometry after 5 hours. Data are from a 1:30 dilution, from 2 experiments, each with 5 per group. All data are shown. *P* values were calculated using a 2-way ANOVA with Dunnett’s multiple-comparison test. Error bars represent the SEM.

Although nucleocapsid-specific antibodies failed to neutralize cell-free SARS-CoV-2 in vitro, we and others have recently shown that nucleocapsid protein is expressed on the surface of SARS-CoV-2–infected cells and therefore has the potential to mediate ADCC (24, 25). To determine whether this could be the mechanism of action in the current study, we performed ADCC assays using sera from mice immunized with the nucleocapsid vaccine. We also examined ADCC activity with the nucleocapsid-specific mAb. Interestingly, we observed that N-specific antibodies from immune sera and N-specific mAbs bound to SARS-CoV-2–infected cells (Figure 7A). Moreover, nucleocapsid-specific sera and nucleocapsid-specific mAbs triggered ADCC activity by NK cells, as evidenced by in vitro CD107a degranulation (Figure 7B). These findings suggest that nucleocapsid-specific antibodies conferred antiviral protection via recognition of infected cells displaying the nucleocapsid antigen on their surface, which triggered Fc-mediated antibody-effector functions.

## Discussion

The SARS-CoV-2 spike protein mediates virus entry by binding to the ACE2 receptor, and, therefore, this protein is considered the most important antigenic target for vaccines and mAb therapies. All approved SARS-CoV-2 vaccines target the spike protein with the goal of generating spike-specific antibodies that block virus entry. However, it is unclear if antibodies of other specificities (e.g., internal viral proteins that do not mediate virus entry) can play a role in antiviral protection. In particular, nucleocapsid-specific antibodies are generated after SARS-CoV-2 infection and also after immunization with experimental nucleocapsid-based vaccines (14, 22), but it is unclear if nucleocapsid-specific antibody responses can confer any protection in vivo.

To the best of our knowledge, the data presented here provide the first demonstration that nucleocapsid-specific humoral responses can help clear a SARS-CoV-2 infection. These findings may be important for the development of next-generation SARS-CoV-2 vaccines encoding other antigens besides the spike protein and for developing new mAb therapies. Current mAb therapies for COVID-19 target only the spike protein, and many of these therapies have lost efficacy against variants, since the spike protein is highly variable. Although the widely distributed Omicron variant exhibits mutations in the nucleocapsid protein, the vast majority of mutations are focused in the spike protein. Similarly, most of the genetic diversity in HIV (and other rapidly mutating viruses) occurs in the envelope proteins that mediate virus entry, motivating the development of mAb therapies that exclusively target these surface viral proteins for the prevention and treatment of HIV infection (26, 27). Our data provide a rationale for the clinical evaluation of mAb therapies targeting internal viral proteins, which may provide another layer of immune protection by engaging antibody effector mechanisms. Although nucleocapsid-specific antibodies do not prevent breakthrough infection with SARS-CoV-2, they help control infection. Following initial infection, virus can be transmitted via cell-to-cell interactions, and this type of infection is resistant to neutralizing antibodies, but not antibody effector mechanisms that target cell-surface antigens (28). It is therefore possible that nucleocapsid-specific mAbs could also synergize with spike-specific mAbs, which are currently a standard of care given either as pre- or post-exposure prophylaxis in high-risk individuals.

In a prior study, we demonstrated that a nucleocapsid-based vaccine confers limited protection against a SARS-CoV-2 challenge when given as a “single vaccine” without a spike-based vaccine. At first glance, this prior study appears to contradict our results, but in that earlier study we used a high challenge dose of SARS-CoV-2 (5 × 10^4^ PFU) and evaluated viral loads during the hyperacute phase of infection (72 hours), which may have been too early to observe virologic differences, given that the efficacy of nucleocapsid-specific antibodies may rely on the recruitment and effector mechanisms of immune cells. The antiviral effects of nucleocapsid-specific antibodies may also vary depending on the virus inoculum. Following low-dose viral challenges (10^3^ PFU), which better represent what humans encounter during a natural infection, nucleocapsid-specific antibodies elicited a 163-fold reduction in viral titers (Figure 6C). However, following high-dose viral challenges (5 × 10^4^ PFU), nucleocapsid-specific antibodies elicited only a 3-fold reduction in viral titers (Figure 2D and Figure 6F). Although the antiviral effect was more limited during the high-dose viral challenge, there was an overall reduction in emaciation and inflammation following treatment with nucleocapsid-specific antibodies.

For more than a century, convalescent plasma therapy has been used to treat many infectious diseases. Convalescent plasma therapy has also shown efficacy against COVID-19, but its benefits are more apparent in patients who receive plasma with high antibody titers, which can only be obtained from convalescent donors early after infection (29, 30). A limitation of our study is that we only performed adoptive sera transfers early after nucleocapsid vaccination using a prime-boost regimen that elicited strong antibody responses. It is unknown whether a similar level of protection would be observed if the passive immunizations were performed using nucleocapsid-immune sera from later time points after vaccination, when antibody titers decline. We detected memory B cells, suggesting that the nucleocapsid-specific antibody response was long lived, but future studies are needed to evaluate the durability of nucleocapsid-specific antibody responses. Recent work from our laboratory and others have shown that the nucleocapsid protein can be expressed on the surface of infected cells and can trigger ADCC (24, 25). Although these studies did not evaluate in vivo viral control, their findings are consistent with our observation that nucleocapsid-specific antibodies could exert a virological effect by engaging effector mechanisms, independent of neutralization. This notion was corroborated by our ADCC studies, which demonstrated that both vaccine-induced and monoclonal nucleocapsid–specific antibodies triggered degranulating activity in NK cells. Taken together, these data provide insights for the development of next-generation vaccines and for expanding the armamentarium of mAb-based therapies for COVID-19 as well as other viral diseases.

## Methods

### Cell lines.

Adenoviral vectors were propagated using HEK293 cells purchased from the American Type Culture Collection (ATCC) (catalog CRL-1573). Vero E6 cells were used to propagate the SARS-CoV-2 isolate USA-WA1/2020 (BEI resources, NR-52281). Cells were not authenticated, as they were purchased from a reputable vendor, and a certificate of analysis was obtained.

### Mice and vaccinations.

Six- to 8-week-old mice were used in these studies. Adult mice, approximately half of which were females and half males, were used for the immunogenicity experiments included in this study. For the challenge studies, female mice were used. Mice were purchased from The Jackson Laboratory and were housed at Northwestern University or the University of Illinois at Chicago (UIC) animal facility. WT C57BL/6 mice were used for immunogenicity studies, and K18-hACE2 mice (on a C57BL/6 background) were used for the challenge studies. These mice express the human ACE2 protein behind the keratin 18 promoter, directing expression in epithelial cells. K18-hACE2 mice were purchased from The Jackson Laboratory (stock no. 034860). Mice were immunized intramuscularly (50 μL per quadriceps) with Ad5 vector expressing SARS-CoV-2 nucleocapsid protein (Ad5-N) at 10^11^ PFU per mouse and N protein, diluted in sterile PBS. Ad5-N was a gift of the Masopust and Vezys laboratory (21). This is a nonreplicating Ad5 vector (E1 and E3 deleted). The vector contains a CMV promoter driving the expression of the respective proteins. The Ad5 vector was propagated on *trans*-complementing HEK293 cells (ATCC), purified by cesium chloride density gradient centrifugation, titrated, and then frozen at −80°C.

### SARS-CoV-2 virus and infections.

SARS-related coronavirus 2, isolate USA-WA1/2020, NR-52281, was deposited by the CDC and obtained through BEI Resources, National Institute of Allergy and Infectious Diseases (NIAID), NIH. Virus was propagated and tittered on Vero-E6 cells (ATCC). In brief, Vero cells were passaged in DMEM with 10% FBS and GlutaMAX (Thermo Fisher Scientific). Cells that were passaged fewer than 20 times were used for all studies. Virus stocks were expanded in Vero-E6 cells following a low MOI (0.01) inoculation and harvested after 4 days. Viral titers were determined by plaque assay on Vero-E6 cells. Viral stocks were used after a single expansion (passage 1) to prevent genetic drift (a small proportion of the virus stock contained mutations in the furin cleavage site). K18-hACE2 mice were anesthetized with isoflurane and challenged intranasally with SARS-CoV-2.

### SARS-CoV-2 RNA quantification.

Lungs were harvested from the infected mice and homogenized in PBS. RNA was isolated with the Zymo 96-well RNA isolation kit (catalog R1052) following the manufacturer’s protocol. SARS-CoV-2 viral burden was measured by RT-qPCR using TaqMan primer and probe sets from Integrated DNA Technologies (IDT) with the following sequences: forward, 5′-GACCCCAAAATCAGCGAAAT-3′, reverse 5′-TCTGGTTACTGCCAGTTGAATCTG-3′; probe 5′-ACCCCGCATTACGTTTGGTGGACC-3′. A SARS-CoV-2 copy number control was obtained from BEI Resources (NR-52358) and used to quantify SARS-CoV-2 genomes.

### FFAs and FRNT assays using live SARS-CoV-2.

Quantification of SARS-CoV-2 by FFA was performed by serial dilution of viral stocks or lung homogenate. Dilutions were added onto a monolayer of Vero cells in a 96-well plate. One hour after infection, cells were overlaid with 1% (w/v) methylcellulose in 2% FBS and 1× MEM. Plates were fixed for 30 minutes with 4% paraformaldehyde (PFA) 24 hours after infection. Staining involved 1° anti–SARS guinea pig serum (1:15,000; NR-10361, BEI Resources) and 2° goat anti–guinea pig HRP (200 ng/mL) in Perm Wash Buffer (0.1% saponin and 0.1% BSA in PBS). Treatment with TrueBlue peroxidase substrate (KPL, LGC Clinical Diagnostics) produced focus-forming units (FFU) that were quantified with an ImmunoSpot ELISpot plate scanner (Cellular Technology). For FRNT assays, serial dilutions of heat-inactivated serum from vaccinated mice were incubated with 100 FFU live SARS-CoV-2 (isolate USA-WA1/2020) for 1 hour at 37°C before infecting a monolayer of Vero cells in a 96-well plate. Viral foci were determined as above for FFAs.

### Reagents, flow cytometry, and equipment.

Single-cell suspensions were obtained from PBMCs or tissues. Dead cells were gated out using LIVE/Dead Fixable Dead Cell Stain (Invitrogen, Thermo Fisher Scientific). SARS-CoV-2 nucleocapsid protein was biotinylated and conjugated to streptavidin-phycoerythrin (PE) for the detection of nucleocapsid-specific memory B cells on magnetic-activated cell sorting–purified (MACS-purified) B cells. MHC class I monomers (D^b^219, LALLLLDRL and K^b^VL8, VNFNFNGL) were used to detect virus-specific CD8^+^ T cells and were obtained from the NIH tetramer facility located at Emory University. MHC monomers were tetramerized in house. Cells were stained with fluorescence-labeled antibodies against CD8α (53-6.7 on PerCP-Cy5.5, BD Pharmingen), CD44 (IM7 on Pacific blue, BioLegend), and K^b^N219 (PE) (BD Pharmingen). Flow cytometry samples were acquired using a BD Canto II or an LSR II and analyzed with FlowJo, version 10 (Tree Star).

### SARS-CoV-2 nucleocapsid–specific ELISA.

Binding antibody titers were quantified by ELISA, as described previously (31, 32), using nucleocapsid protein as a coating antigen. In brief, MaxiSorp 96-well, flat-bottomed plates (Thermo Fisher Scientific) were coated with SARS-CoV-2 nucleocapsid protein and then washed and blocked. Serial sera dilutions were performed. Absorbance was measured using a SpectraMax Plus 384 (Molecular Devices). Antibody levels were reported as endpoint titers using serial 3-fold dilutions.

### Sera transfers and mAb therapies.

For the passive immunization studies, 500 μL N-specific immune sera or irrelevant sera were transferred 1 day before SARS-CoV-2 challenge. Control mAb (IgG2a, clone C1.18.4) and anti-N mAb (clone 1C7C7) were purchased from Leinco. Mice received 800 μg of the respective antibody clone diluted in PBS 1 day before SARS-CoV-2 challenge. Sera transfers and mAb therapy were administered intraperitoneally.

### ADCC assays.

293-ACE2 cells were made by transducing 293 cells with a lentivirus expressing human ACE2 and selected in blasticidin for 7 days, as previously described (33). Effector NKL cells expressing mouse CD16 (NKL-mCD16) were a gift from Oscar A. Aguilar and Lewis Lanier (UCSF, San Francisco, California, USA) and were maintained as previously described (34). In brief, 293-ACE2 cells were mock-infected or infected with SARS-CoV-2 (England2 strain) at a MOI of 5. After 24 hours, 293-ACE2 cells (25,000/well) were cultured with effectors (NKL-mCD16, 50,000/well), together with sera or mAb (1C7) in the presence of CD107a-FITC (BioLegend) and Golgi-Stop (BD Biosciences). Five hours later, the cultures were washed and stained with LIVE/Dead Fixable Aqua and CD56-BV605 (BioLegend). CD107a levels were analyzed on an Attune flow cytometer (Thermo Fisher Scientific). Cells were gated on the live/CD56^+^ population, and the percentage of CD107a^+^ cells was calculated. All sera and mAb samples were incubated with both infected and mock-infected cells to control for nonspecific activation. Samples were tested over a 3-fold dilution series beginning at 1:30 to account for “hooking” effects. Every sample was tested in technical duplicate and averaged.

### Detection of N-specific antibody binding against infected cells.

293-ACE2 cells were infected with SARS-CoV-2 (England2 strain) at a MOI of 5. After 24 hours, cells were detached with TrypLE (Gibco, Thermo Fisher Scientific) and stained with primary N-specific mAb (or N-specific immune sera), as indicated, for 30 minutes at 4°C. After washing, cells were incubated with a secondary antibody (anti–mouse Alexa Fluor 647 (Thermo Fisher Scientific) for 30 minutes at 4°C. Cells were washed, fixed in 4% PFA, and analyzed on an Attune flow cytometer.

### Statistics.

A 2-tailed Mann-Whitney *U* test or 2-way ANOVA was used to determine statistical significance. All data represent the mean ± SEM. Dashed lines in the data figures indicate the limit of detection. Statistical significance was established at a *P* value of 0.05 or less and was generally assessed by Mann-Whitney *U* test, unless indicated otherwise in the figure legends. Data were analyzed using GraphPad Prism (GraphPad Software).

### Study approval.

Human specimens. All protocols used for participant recruitment, enrollment, blood collection, sample processing, and immunological assays with human samples were approved by the IRB of Northwestern University (STU00212583). All participants voluntarily enrolled in the study by signing an informed consent form after receiving detailed information about the clinical study. All procedures involving animals were performed with the approval of the Center for Comparative Medicine at Northwestern University and the IACUC of UIC. Mouse infections were performed at the UIC following Biosafety Level 3 (BSL-3) guidelines with approval of the IACUC of UIC.

## Author contributions

TD and SS performed the vaccination and immunogenicity experiments. JC performed the challenge experiments. YRC helped with the H&E studies. KB and RJS designed and performed the ADCC assays. PPM and JMR designed the immunogenicity and challenge experiments and secured funding. PPM wrote the manuscript, with feedback from all authors. IJK and LV obtained the IRB and consent forms and collected human samples for immunological assays.

## Supplementary Material

Supplemental data

## Version 1 10/11/2022

In-Press Preview

## Version 2 12/01/2022

Electronic publication
