# Supplementary material for: Improved control of SARS-CoV-2 by treatment with a nucleocapsid-specific monoclonal antibody
Source: J Clin Invest. 2022 Dec 1;132(23):e162282. doi: 10.1172/JCI162282 (PMC9711872; doi:10.1172/JCI162282)
Supplement: Supplemental data [file jci-132-162282-s074.pdf]

Improved control of SARS-CoV-2 by treatment with nucleocapsid-specific monoclonal antibody

Tanushree Dangi[1], Sarah Sanchez[1], Jacob Class[2], Michelle Richner[2], Lavanya Visvabharathy[3], Young Rock Chung[1], Kirsten Bentley[4], Richard J. Stanton[4], Igor J. Koralnik[3], Justin M. Richner*[2], Pablo Penaloza-MacMaster*[1]

[1]Department of Microbiology and Immunology, Northwestern University Feinberg School of Medicine, Chicago, IL 60611. [2]Department of Microbiology & Immunology, University of Illinois at Chicago College of Medicine, Chicago, IL 60612. [3]Ken and Ruth Davee Department of Neurology, Feinberg School of Medicine, Northwestern University, Chicago, Illinois, USA. [4]Division of Infection and Immunity, School of Medicine, Cardiff University, Cardiff, United Kingdom.

***Correspondence and Lead contacts:**

Justin Richner (richner@uic.edu) & Pablo Penaloza-MacMaster (ppm@northwestern.edu)

Supplemental Figures:

Figure S1

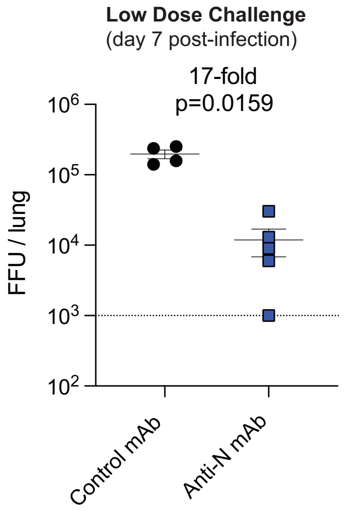

Figure S1. Nucleocapsid-specific monoclonal antibody (mAb) improves the control of SARS-CoV-2 infection. Viral loads in lungs by focus forming assays. These data are from the same mice from Figure 6A-6C, except that viral loads in lungs were quantified by focus forming assays. Challenges were performed with a total of 4-5 mice per group in Biosafety level 3 (BSL-3) facilities. Indicated P-values were calculated using Mann Whitney test.

Figure S2

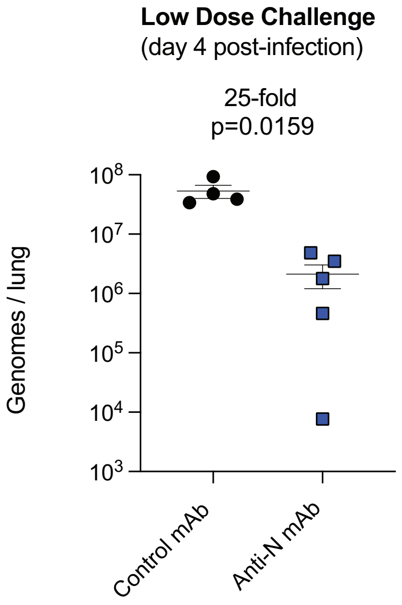

Figure S2. Nucleocapsid-specific monoclonal antibody (mAb) improves the acute control of SARS-CoV-2 infection. Viral loads in lungs by RT-qPCR. Experiment was identical to that of Figure 6A, except that mice were euthanized after 4 days. Challenges were performed with a total of 4-5 mice per group in Biosafety level 3 (BSL-3) facilities. Indicated P-values were calculated using Mann Whitney test.

Figure S3

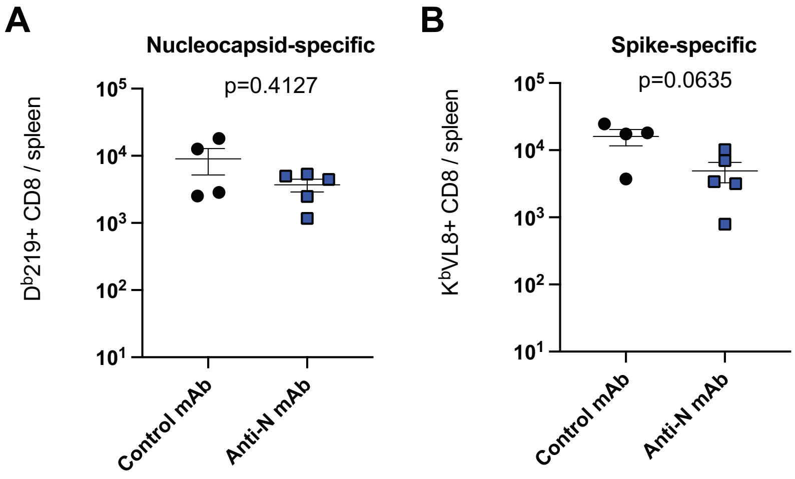

Figure S3. Nucleocapsid-specific monoclonal antibody (mAb) does not significantly affect virus-specific CD8 T cell responses. (A) Numbers of SARS-CoV-2 nucleocapsid-specific CD8 T cells. **(B)** Numbers of SARS-CoV-2 spike-specific CD8 T cells. These data are from the same mice from Figure 6A-6C. Data are from spleen at day 7 post-infection. Challenges were performed with a total of 4-5 mice per group in Biosafety level 3 (BSL-3) facilities. Indicated P-values were calculated using Mann Whitney test.
